# Genetic diversity of *Coxiella burnetii* in domestic ruminants in central Italy

**DOI:** 10.1186/s12917-018-1499-8

**Published:** 2018-05-29

**Authors:** M. Di Domenico, V. Curini, V. Di Lollo, M. Massimini, L. Di Gialleonardo, A. Franco, A. Caprioli, A. Battisti, C. Cammà

**Affiliations:** 1 Istituto Zooprofilattico Sperimentale dell’Abruzzo e del Molise “G. Caporale”, Campo Boario, 64100 Teramo, Italy; 2 Istituto Zooprofilattico Sperimentale del Lazio e della Toscana “M. Aleandri”, Via Appia Nuova 1411, 00178 Roma, Italy

**Keywords:** *Coxiella burnetii*, Genotyping, MST, MLVA, Italy

## Abstract

**Background:**

As the epidemiology of human Q Fever generally reflects the spread of *Coxiella burnetii* in ruminant livestock, molecular characterization of strains is essential to prevent human outbreaks. In this study we report the genetic diversity of *C. burnetii* in central Italy accomplished by MST and MLVA-6 on biological samples from 20 goat, sheep and cow farms.

**Results:**

Five MST and ten MLVA profiles emerged from the analysis establishing a part of *C. burnetii* strain world atlas. In particular, ST32 occurred on 12 farms (60%), prevalently in goat specimens, while ST12 (25%) was detected on 4 sheep and 1 goat samples. ST8 and a variant of this genotype were described on 2 different sheep farms, whereas ST55 was observed on a goat farm. Five complete MLVA profiles different from any other published genotypes were described in this study in addition to 15 MLVA incomplete panels. Despite this, polymorphic markers Ms23, Ms24 and Ms33 enabled the identification of samples sharing the same MST profile.

**Conclusions:**

Integration of such data in international databases can be of further help in the attempt of building a global phylogeny and epidemiology of Q fever in animals, with a “One Health” perspective.

## Background

*Coxiella burnetii* is the causative agent of Query (Q) fever in humans, a zoonotic disease present throughout the world, except in New Zealand [1], and coxiellosis in animals [2]. It is an obligate intracellular bacterium, replicating in eukaryotic cells that shows a biphasic developmental cycle: the large cell variant (LCV) corresponding to the intracellular replicative form and the small cell variant (SCV) which represents the host cell free stable form that is highly resistant to different environmental stresses [3]. The SCVs may persist in the environment for years [1, 3]. *C. burnetii* is found in urine, faeces and milk of infected animals, although transmission to humans is most frequently due to the inhalation of aerosolized bacteria that are spread in the environment by infected animals after delivery or abortion. Amniotic fluid and placenta contain the highest concentration of bacteria [4]. In recent years, an increasing number of animals have been reported to shed the bacterium, including pets, reptiles, ticks, rodents and birds [5–8], however, the main reservoirs of *C. burnetii* are cattle, sheep, and goats [9, 10]. Because Q fever is a zoonosis, the epidemiology of human infections generally reflects the circulation of the bacterium in ruminant livestock and serological investigation. Prophylaxis and molecular characterization of the strains circulating is therefore essential in order to prevent human outbreaks. Several genotyping methods have been described thus far. Until 2005, these techniques were based on plasmid typing, restriction fragment length polymorphism followed by pulsed-field gel electrophoresis (RFLP-PFGE) analysis, and sequence analysis of individual genes (e.g. *16S*). Nevertheless, some of these methods exhibited limitations on inter- and intra-laboratory reproducibility and poor discrimination power that hindered their widespread use [11]. In 2005, typing via sequence analysis of multi-spacer regions (MST) was introduced by Glazunova et al. [12], who identified 10 highly variable intergenic spacers allowing the unambiguous characterization of the agent. MST genotyping has high levels of discrimination and helps to trace the spread of *C. burnetii* from one region to another and to define phylogenetic relationships [13, 14]. Otherwise, multi-locus Variable Number Tandem Repeat (VNTR) analysis (MLVA) was first established by Svraka et al. [15], and then improved by Arricau-Bouvery et al. [16]. Since their rapid evolution VNTR are extremely polymorphic, therefore MLVA usually provide a better discriminatory power, which is often suitable for epidemiological purposes [17]. MST and MLVA are both PCR-based techniques, and they have the potential to be used directly on non-cultured samples, avoiding the culture step of the pathogen that requires biosafety level 3 and long-time analyses. In this study, we report the genetic diversity of *C. burnetii* by MST and MLVA with the aim of describing the strains circulating in central Italy, taking into account the knowledge acquired after the Q Fever outbreaks in the Netherlands [18–20].

## Methods

### Biological samples and DNA extraction

**Fig. 1:**
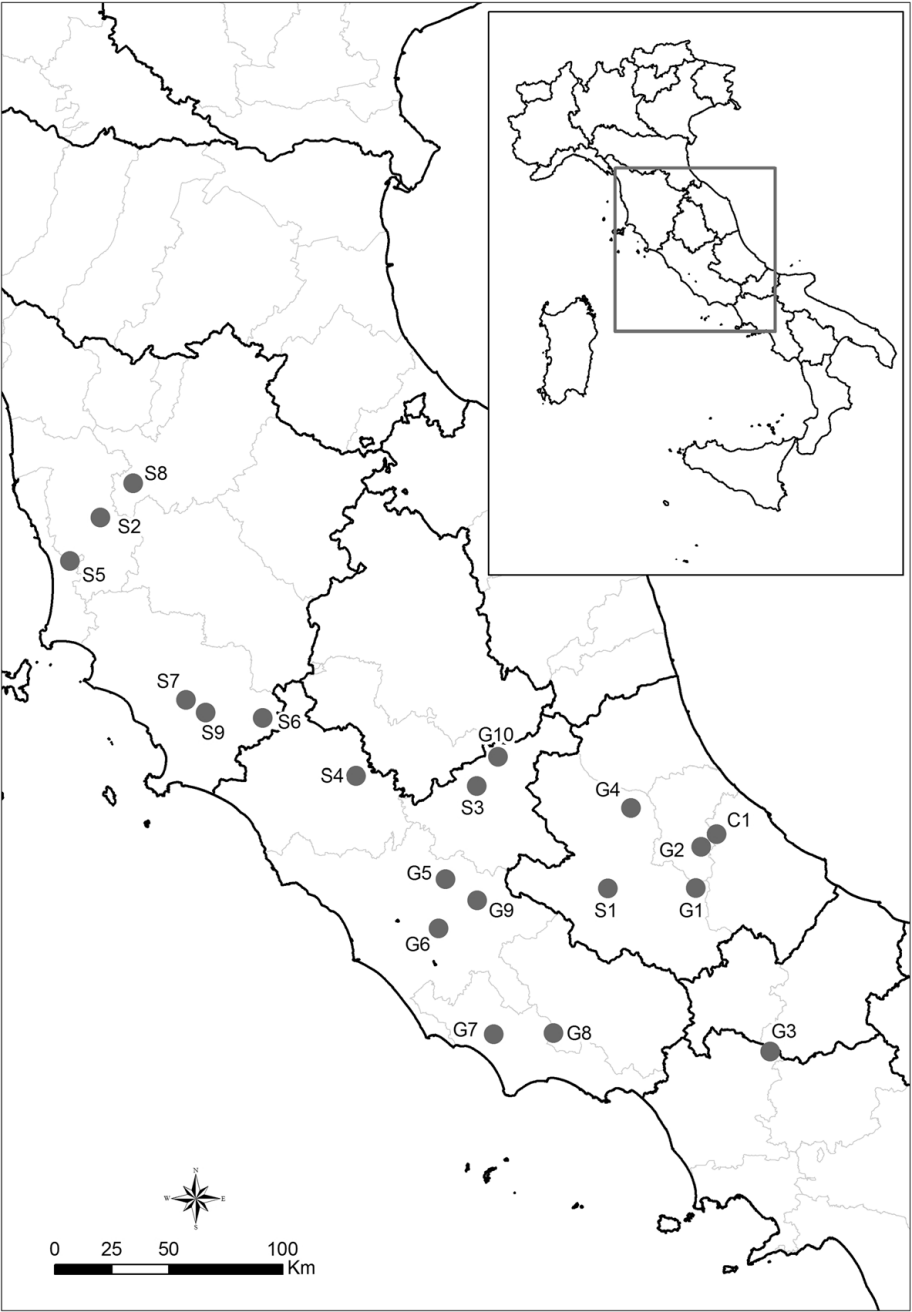
Geographical localization of the farms in central Italy. Farm ID was designated according to animal species host: G = goat; S = sheep; C = cattle

During the period 2012–2015 20 farms were positive for *C. burnetii* by Real Time PCR [21], with slight modifications [14]. Farms were named G1-G10 for goat, S1-S9 for sheep, and C1 for cow (Table 2). Brain, spleen, lung and liver were sampled from aborted goat (N. 10) and sheep (N. 9) fetuses and vaginal swabs were collected from the relative parturient animals. Placenta was collected only in two sampling sessions, these samples referred to a goat (G2) and sheep (S9). A milk sample was collected from a serological positive cow (C1). Only one DNA sample per farm was used for genotyping purposes; the selection was driven by the lowest C_t_ values obtained by Real Time PCR. Details of the samples per geographic origin, specimen, year of collection and C_t_ value are reported in Table 1 and Fig. 1.

**Table 1:** Detailed information of the specimens

ID^a^	Province	Year	Species	Specimen	Ct value
G1	Pescara	2015	goat	brain	31
G2	Pescara	2014	goat	placenta	8
G3	Caserta	2015	goat	lung	28
G4	L’Aquila	2015	goat	brain	28
C1	Chieti	2016	cattle	milk	23
S1	L’Aquila	2015	sheep	brain	29
S2	Pisa	2012	sheep	vaginal swab	21
S3	Rieti	2015	sheep	spleen	29
S4	Viterbo	2014	sheep	lung	30
G5	Roma	2013	goat	vaginal swab	20
G6	Roma	2012	goat	spleen	26
G7	Latina	2014	goat	liver	19
G8	Latina	2014	goat	spleen	24
G9	Roma	2014	goat	lung	22
S5	Livorno	2014	sheep	spleen	22
S6	Grosseto	2015	sheep	lung	23
S7	Grosseto	2015	sheep	spleen	24
S8	Firenze	2013	sheep	vaginal swab	22
S9	Grosseto	2015	sheep	placenta	28
G10	Rieti	2014	goat	lung	26

^a^Farm ID designation according to animal species host: *G* goat, *S* sheep, *C* cattle. Samples per farm with the lowest Ct values were reported only

DNA from fetal organs was isolated using the Maxwell 16 Tissue DNA Purification Kit (Promega) following the manufacturer’s instructions. Cells from milk were recovered by centrifuging 50 ml at 2000 g for 10 min; the pellet was resuspended with 300 μl of nuclease free water and then DNA was isolated using the Maxwell 16 Cell DNA Purification Kit (Promega) following the manufacturer’s instructions.

### MST and MLVA

Multispacer Sequence Typing of *C. burnetii* DNA was performed on 20 specimens as previously described by Glazunova et al. [12] with some modifications [14]. Raw sequence data were assembled using SeqMan Pro (DNASTAR Lasergene 10 Core Suite). The coded alleles were compared with the sequences in the reference database available on the website http://ifr48.timone.univ-mrs.fr/mst/coxiella_burnetii/groups.html. MLVA was performed on the same samples for the 6 loci panel [16] with some modifications described by Tilburg et al. [22]. Furthermore, in order to obtain complete panels, new reverse primers were designed for the refractive markers Ms24 (5’-ACAAGCTATTTACTCCCTTTCTGC-3′) and Ms34 (5’-GCGTTAGTGTGCTTATCTCTTG-3′) by the Primer Express v3.0.1 software (Applied Biosystems), while for Ms23 and Ms33 primers from the website http://mlva.u-psud.fr/MLVAnet/spip.php?rubrique50 were selected. Three more DNA samples for which MST profile was previously reported [14], were included in the VNTR analysis for a total of 23 different strains. The amplification products were diluted 1:10 and 1:100 in distilled water and 1.2 μl of every single dilution was added to reaction mixture containing 10.5 μl formamide and 0.3 μl of Gene Scan 500 size standard marker (Applied Biosystems). PCR products were denatured for 30 s at 95 °C, cooled on ice for 2 min and then run on AB3130XL capillary sequencer with POP7 polymer. VNTR fragments were finally sized via GeneMapper software v.4.0 (Applied Biosystems). DNA from the Nine Mile strain (RSA 493) was used as a reference. According to the online database support site (http://mlva.u-psud.fr/mlvav4/genotyping/index.php), the MLVA profile of the Nine Mile strain is 9 27 4 6 9 5 for markers Ms23, Ms24, Ms27, Ms28, Ms33 and Ms34, respectively. For each marker the repeats number was determined inferring the sizes of the sample fragments with those obtained using the reference strain, run in the same time. Ten samples were sequenced to confirm the repeats number and the presence of Insertion Sequences (IS).

## Results

**Fig. 2:**
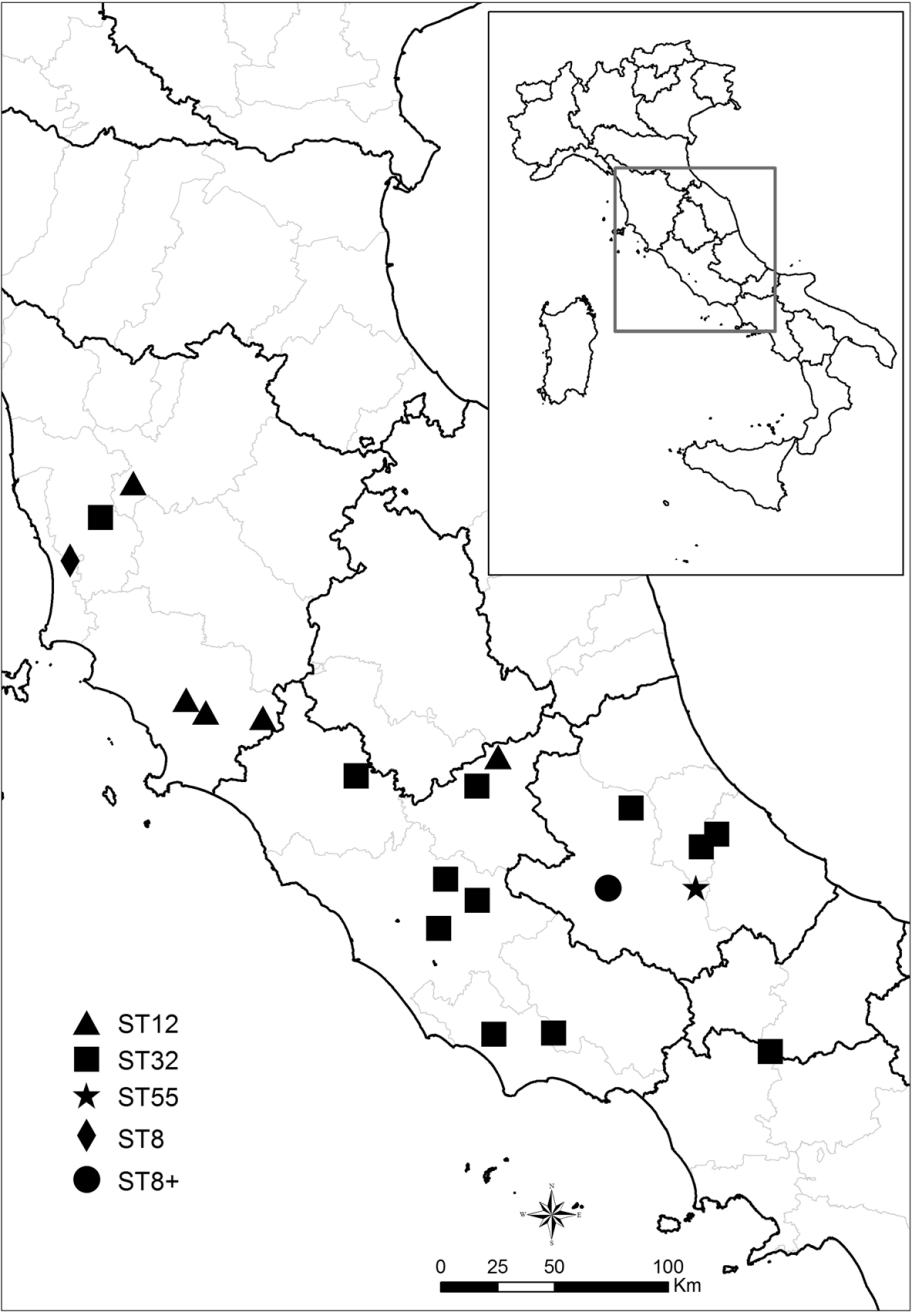
Genetic diversity. Sequence types are expressed accordingly to MST database. ST8+ refers to a variant of the ST8 as previously described (Di Domenico et al. [14])

The MST analysis from the 10 goat, 9 sheep and 1 cattle PCR-positive farms revealed two dominant previously described Sequence Types: ST32, reported in 12 farms (60%), and ST12, reported in 5 farms (25%). In addition, ST8 and ST55 were reported in the farms S5 (5%) and G1 (5%) respectively. Finally, a variant of the ST8 (proposed ST62) previously described only in this geographic area [14], was described in farm S1 (5%). This new allelic combination is 5 4 2 5 1 5 3 2 4 4 for the spacers Cox2, Cox5, Cox18, Cox20, Cox22, Cox37, Cox51, Cox56, Cox57, and Cox61 respectively. A complete panel was accomplished for all samples through MST analysis (Table 2, Fig. 2), while incomplete results were obtained for MLVA (Table 2). Although only partial panels were gained for the samples G1, G3-G6, G8-G10, S1-S5, S9 and C1, the high resolution power of MLVA allowed differentiation of strains sharing the same MST profile such as the case of the sample from farm G3, with ST32 like eight other farms, but showing a unique allele (12 tandem repeats) in the Ms24 marker. The case of strains from goat farms G7 and G8, located in the same geographic area and having the same ST32, is analogous. The variation in the number of tandem repeats in the locus Ms24 allowed the differentiation of the two genotypes showing 11 and 10 tandem repeats respectively, as well as the strains from farms S9 and G10 (11 and 12 tandem repeats, respectively), while showing the same ST12. Moreover, the polymorphism of the marker MS33 allows the discrimination of the strain G8 (9 tandem repeats) from G2 and G9 (5 tandem repeats + IS1111), all of these sharing the same ST32. Ten different MLVA genotypes emerged from the analysis (Table 2). Three samples are omitted from the MLVA description because of the limited amount of DNA available. The five complete MLVA profiles compared with those deposited in the public MLVA database (http://mlva.u-psud.fr/mlvav4/genotyping/index.php) did not match with any of the published genotypes. Sequence analysis of the samples G2, G7, and G9 confirmed the presence of the IS1111.

**Table 2:** MST and MLVA genotyping of *C. burnetii* DNA from domestic ruminants in central Italy

Farm ID^a^	Province	Specimen	MST	MLVA 6 panel						Study MLVA genotype
				Ms23	Ms24	Ms27	Ms28	Ms33	Ms34	
G1	Pescara	brain	ST55			4	5	6		1
G2	Pescara	placenta	ST32	9	10	2	3	5 (IS1111)^b^	3	2
G3	Caserta	lung	ST32		12	2	3		3	3
G4	L’Aquila	brain	ST32		10	2	3		3	
C1	Chieti	individual milk	ST32	5	10	2	3		3	
S1	L’Aquila	brain	ST8^c^			4	5	8	2	4
S2	Pisa	vaginal swab	ST32		10	2	3		3	
S3	Rieti	spleen	ST32	10		2	3		3	
S4	Viterbo	lung	ST32			2	3		3	
G5	Roma	vaginal swab	ST32		10	2	3		3	
G6	Roma	spleen	ST32		10	2	3		3	
G7	Latina	liver	ST32	8	11	2	3	5 (IS1111)^b^	3	5
G8	Latina	spleen	ST32		10	2	3	9	3	6
G9	Roma	lung	ST32		10	2	3	5 (IS1111)^b^	3	
S5	Livorno	spleen	ST8		7	4	3	8	2	7
S6	Grosseto	lung	ST12							
S7	Grosseto	spleen	ST12							
S8	Firenze	vaginal swab	ST12							
S9	Grosseto	placenta	ST12		11	2	3		3	
G10	Rieti	lung	ST12		12				3	
Di Domenico et al. 2014 (C2) [14]	Pescara	individual milk	ST20^c^	6	12	2	7	6	9	8
Di Domenico et al. 2014 (G11) [14]	L’Aquila	individual milk	ST55	9	22	4	1	3	1	9
Di Domenico et al. 2014 (G12) [14]	L’Aquila	brain	ST8^c^	9	8	4	1	5	2	10

^a^Farm ID designation according to animal species host: *G* goat, *S* sheep, *C* cattle

^b^The presence of the Insertion Sequence IS1111 was confirmed by sequencing

^c^New genotypes described by Di Domenico et al. [14]

## Discussion

Discrimination of bacterial strains based on the analysis of their genetic content has become widely used due to its high resolution. Genetic variability can explain most of the phenotypic heterogeneity within bacterial population, such as host specificity, pathogenicity, antibiotic resistance, and virulence [23]. Currently, two main discriminant methods are commonly used for *Coxiella burnetii* genotyping: MST and MLVA [4]. MST has succeeded in establishing reliable correlations of STs with geographic distribution, clinical manifestations, and epidemiology of strains. Despite some STs being found worldwide, many others are restricted to specific areas. As a result, it has also been qualified as a “geotyping method” [4]. For example, to date ST17 has been isolated only from French Guiana, where it causes severe forms of the disease [24]. Acute Q Fever patients from French Guiana demonstrate significantly higher phase I IgG titers, whereas phase II antibodies are generally revealed at this stage of the disease [4]. Of note, pneumonia is observed in 83% of the patients representing the genotype with the highest prevalence of community-acquired pneumonia in the world [4, 25]. Analogously, ST33 is distributed in different small areas of Europe including Germany, where it was first described, and the Netherlands where it spread via France, causing the largest Q Fever outbreak ever described [26, 27]. Strains belonging to the ST23 group were reported in ticks, birds, ruminants and humans only in a restricted area between Eastern Europe (Czech Republic and Slovakia) and Asian countries (Russia, Kazakhstan, Mongolia and Uzbekistan), (http://ifr48.timone.univ-mrs.fr/mst/coxiella_burnetii/strains.html). Another interesting case is that of ST21. Despite two isolates from France and one from the United States, it is mainly reported in Nova Scotia [12]. On the other hand, some sequence types are distributed worldwide, such as ST16.

In Italy, sequence types already described are ST16, ST18 and ST29 (http://ifr48.timone.univmrs.fr/mst/coxiella_burnetii/strains.html), a novel sequence type similar to ST20, an additional sequence type related to ST8 and ST55 discovered in bovine milk, goat fetus and goat milk, respectively [14]. ST12 and ST32 are closely related on the basis of phylogenetic analysis [13, 14] and largely distributed along the area considered in the present study. Indeed, these two genotypes recur in 85% of the specimens (17/20) in cow, sheep and goat. These findings confirmed the spread of these genotypes in Tuscany as previously reported [28]. Interestingly, ST12 has been detected in clinical human samples from France, Switzerland and Senegal (http://ifr48.timone.univmrs.fr/mst/coxiella_burnetii/strains.html), whereas in animals it has been only found in Italy [28]. This result suggests that goat and sheep could represent an important source of human Q fever in this country. Although the oral exposure is still controversial [9], the transmission of *C. burnetii* to humans through inhalation of contaminated aerosol is widely recognized, especially for certain at-risk categories, such as farmers, veterinarians, or people living close to or exposed to infected flocks [29–32]. Conversely, the zoonotic origin of ST32 and the transmission to human have been already assessed. Indeed, as reported in the database http://ifr48.timone.univ-mrs.fr/mst/coxiella_burnetii/strains.html, it was identified in a goat placenta in Austria, and detected in human heart valve and aortic biopsy in Germany and France, respectively. Our study confirms ST32 detection in sheep specimens and it firstly describes the occurrence of this zoonotic genotype in cows. Three additional MST profiles have been reported in the present study: ST8, ST55 and a novel ST closely related to the ST8 previously identified in the same area [14]. Notably, ST8 was responsible for two human chronic Q Fever cases in Portugal [33].

The Q Fever outbreak in the Netherlands pushed toward the molecular investigation by both MST and MLVA of *C. burnetii* strains in different countries, not only in Europe. As VNTRs are important source of genetic polymorphisms for strain typing due to their rapid evolution, MLVA approach is particularly useful for epidemiological purposes. Unfortunately, PCR amplification is not always successful for all markers, so that partial genotypes are frequently obtained causing underestimation of the genotypic diversity [33–36]. Moreover, insertions or deletions may impair the estimation of the number of tandem repeats [37]. In particular, Ms23 and Ms33 both harbour a recognition site for the IS1111 insertion sequence upstream the repeat units and therefore may constitute preferred targets for insertions [37].

In the present study, five complete MLVA profiles were gained, all of which were different from the previously reported genotypes, including those recently found in Italy [35], while 15 panels were incomplete. Despite this, MLVA enabled 10 genotypes to be identified, instead of the 5 obtained by MST. Moreover, by means of sequencing we detected the presence of the IS1111 within the repetitive region of the Ms33 marker in three different goat samples sharing the same sequence type (ST32).

## Conclusions

Our study based on MST and MLVA-6, established a part of *C. burnetii* strain atlas in central Italy. Integration of such data with international databases can be of further help in the attempt of building a global phylogeny and epidemiology of Q fever in animals, with a “One Health” perspective.

## Abbreviations

LCV: Large cell variant
MLVA: Multi Locus VNTR Analysis
MST: Multispacer Sequence Typing
RFLP-PFGE: Restriction fragment length polymorphism followed by pulsed-field gel electrophoresis
SCV: Small cell variant
ST: Sequence Type
VNTR: Variable Number Tandem Repeat
